# Investigating the Role of Endothelial Glycogen Synthase Kinase3α/β in Atherogenesis in Low Density Lipoprotein Receptor Knockout Mice

**DOI:** 10.3390/ijms232314780

**Published:** 2022-11-26

**Authors:** Lauren Mastrogiacomo, Geoff H. Werstuck

**Affiliations:** 1 Thrombosis and Atherosclerosis Research Institute, 237 Barton Street East, Hamilton, ON L8L 2X2, Canada; 2 Department of Medicine, McMaster University, 1200 Main St. W, Hamilton, ON L8N 3Z5, Canada

**Keywords:** cardiovascular disease, glycogen synthase kinase3, atherosclerosis, endothelial activation

## Abstract

Risk factors for developing cardiovascular disease (CVD) are associated with inflammation and endothelial activation. Activated endothelial cells (ECs) express adhesion proteins that recruit monocytes to the subendothelial layer initiating plaque development. Understanding the mechanism(s) by which ECs increase adhesion protein expression will facilitate the development of therapies aimed at preventing CVD progression and mortality. Glycogen synthase kinase (GSK)3α/β are constitutively active kinases which have been associated with many cellular pathways regulating cell viability and metabolism. While roles for myeloid GSK3α/β in the development of atherosclerosis have been established, there is limited knowledge on the potential roles of endothelial GSK3α/β. With the use of Cre recombinase technology, GSK3α/β was knocked out of both ECs and macrophages (Tie2Cre GSK3α/β^fl/fl^ LDLR^−/−^). A bone marrow transplant was used to replenish GSK3α/β in the myeloid lineage allowing the assessment of an endothelial-selective GSK3α/β knockout (BMT Tie2Cre GSK3α/β^fl/fl^ LDLR^−/−^). In both models, adhesion protein expression, macrophage recruitment and plaque volume were reduced in GSK3α knockout mice. GSK3β knockout had no significant effect. Results from this study are the first to suggest a pro-atherogenic role of endothelial GSK3α and support existing evidence for targeting GSK3α in the treatment of atherosclerotic CVD.

## 1. Introduction

Atherosclerosis is a multifactorial inflammatory disease that involves accumulation of lipids and inflammatory cells in the subendothelial layer, causing a narrowing of the artery, altering vascular blood flow, and increasing risk of thrombus formation and/or cardiac complications [1,2,3,4]. Atherosclerotic CVD is the leading cause of mortality worldwide [5,6]. Although effective, current medications target specific risk factors, such as hypertension or dyslipidemia, rather than the disease process itself [7,8]. Understanding the mechanism(s) underlying the initiation and development of atherosclerosis will facilitate the development of more targeted therapies to prevent or reduce the risk for CVD.

The earliest detectable stage in atherogenesis is endothelial activation [9]. The endothelium forms a single layer of semi-permeable cells that line the vasculature that performs critical functions in the maintenance of vascular homeostasis [10]. Risk factors for CVD, including hyperglycemia, hypertension, dyslipidemia and smoking, are each associated with arterial inflammation and activation of the EC layer [11]. EC activation is characterized by expression of leukocyte adhesion proteins, including P-selectin, E-selectin, ICAM-1 and VCAM-1 [12]. These adhesion proteins facilitate the recruitment of circulating monocytes to the injured endothelium. Monocytes migrate into the subendothelial layer where they differentiate into macrophages. These macrophages endocytose lipids and cellular debris, becoming lipid engorged foam cells [13,14]. Understanding the mechanism underlying adhesion protein expression could potentially reveal novel therapeutic targets to halt atherogenesis and plaque progression.

GSK3 is an enzyme involved in many cellular signalling pathways [15]. There are two isoforms of GSK3, GSK3α and GSK3β that are 98% homologous in the kinase domain [16]. These isoforms are now known to possess both redundant and isoform-specific functions [17]. A whole body GSK3α knockout mouse model is viable, however a whole body GSK3β knockout mouse dies during mid-gestation [18,19]. Previous research has indicated that a whole body GSK3α knockout attenuates atherosclerosis in mice [20]. Further research has shown that a myeloid-specific knockout of GSK3α can also attenuate atherosclerosis, however not to the extent observed in the whole body GSK3α knockout mouse [21]. These findings suggest that GSK3α plays a pro-atherosclerotic role in other cells of the body.

ECs play a critical role in the initiation of atherosclerosis and little is known about the potential role of GSK3α/β in the endothelium. In this study, Cre-Lox technology was used to create an endothelial GSK3 knockout in LDLR^−/−^ mice in order to investigate the potential role of endothelial GSKα/β in atherogenesis.

## 2. Results

### 2.1. The Tie2Cre Recombinase Is Not Endothelial Specific

In this study, Tie2Cre GSK3α^fl/fl^ LDLR^−/−^ and Tie2Cre GSK3β^fl/fl^ LDLR^−/−^ mice were created in order to investigate the endothelial-specific role(s) of GSK3α and GSK3β. It has previously been reported that the Tie2Cre mouse strain (B6.Cg-Tg (Tek-cre)1Ywa/J) may express Cre recombinase activity in some myeloid lineages [22]. Previous work from our lab has found that deletion of GSK3α in macrophages attenuated atherosclerosis in LDLR^−/−^ mice [21]. Therefore, it was important to determine the specificity of GSK3α/β knockout in our Tie2Cre strains. Peritoneal macrophages and ECs were isolated from GSK3α^fl/fl^ LDLR^−/−^, GSK3β^fl/fl^ LDLR^−/−^, Tie2Cre GSK3α^fl/fl^ LDLR^−/−^ and Tie2Cre GSK3β^fl/fl^ LDLR^−/−^ mice and the gene expression of GSK3α or GSK3β was analysed. RT-PCR analysis showed that macrophages and ECs from Tie2Cre GSK3α^fl/fl^ LDLR^−/−^ showed a significant decrease in GSK3α, but not GSK3β, expression, compared to GSK3α^fl/fl^ LDLR^−/−^ mice (Supplementary Figure S1A). No significant differences were observed in GSK3α or GSK3β expression in the lungs, liver, or skeletal muscle. RT-PCR analysis from Tie2Cre GSK3β^fl/fl^ LDLR^−/−^ mice showed a significant decrease in macrophage and endothelial GSK3β, but not GSK3α, compared to GSK3β^fl/fl^ LDLR^−/−^ mice (Supplementary Figure S1B). No significant differences were observed in gene expression in lung, liver or skeletal muscle. These results indicate that, in these Tie2Cre strains, GSK3 is affected in macrophages, as well as ECs. Therefore, this is not an endothelial specific model. This mouse model will be referred to hereon as an endothelial/macrophage knockout of GSK3α or GSK3β.

### 2.2. Endothelial/Macrophage Knockout of GSK3α Attenuates Atherosclerosis

To determine the effect of an endothelial/macrophage GSK3α or GSK3β knockout on atherogenesis, mice were placed on a HFD to initiate plaque formation. After 3 weeks of HFD feeding, no significant differences were observed in plasma lipids or body weight of Tie2Cre GSK3α^fl/fl^ LDLR^−/−^ mice, compared to age matched LDLR^−/−^ or Tie2Cre LDLR^−/−^ controls (Table 1). Mice carrying the GSK3β^fl/fl^ allele tended to present with elevated plasma triglycerides, body weight and adipose weight, relative to Tie2Cre LDLR^−/−^ control. This appears to be a result of non-GSK3 related differences in this strain as the effect was independent of Cre recombinase activity.

Plaque volumes were visualized and quantified using Masson’s trichrome staining. LDLR^−/−^, Tie2Cre LDLR^−/−^, GSK3α^fl/fl^ LDLR^−/−^ and GSK3β^fl/fl^ LDLR^−/−^ mice showed no difference in plaque volume, suggesting that neither the Tie2Cre recombinase nor GSK3α^fl/fl^/GSK3β^fl/fl^ had an effect on atherosclerotic plaque formation (Supplementary Figure S2). GSK3α^fl/fl^ LDLR^−/−^ and GSK3β^fl/fl^ LDLR^−/−^ mice, lacking the Tie2Cre recombinase, were chosen as controls for the remainder of this study, as they are littermates with the experimental mice.

**Figure 1:**
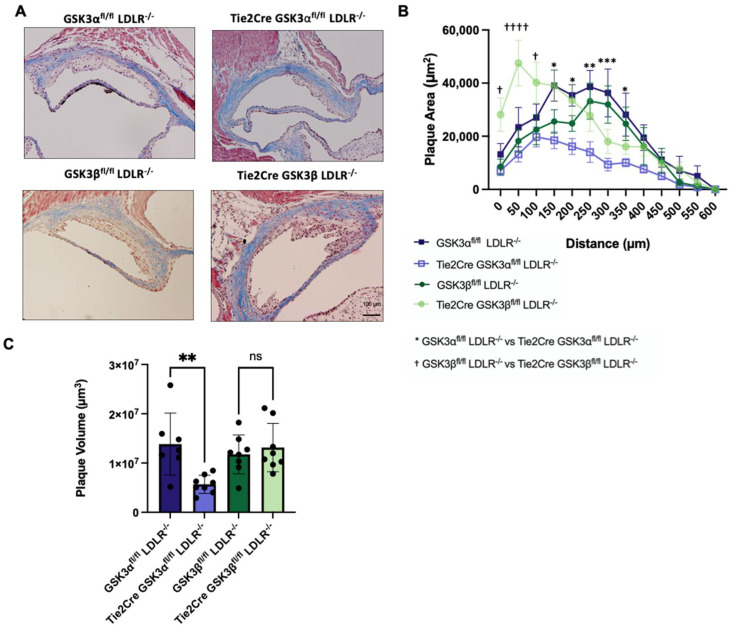
Atherosclerosis in endothelial/macrophage GSK3α/β knockout mice. (**A**) Representative images of Masson’s trichrome staining of cross sections of aortic sinus from 15 week old control or endothelial/macrophage GSK3α/β knockout mice. Scale bar represents 100 μm. (**B**) Quantification of plaque area at the aortic sinus of the 15 week old mice. (**C**) Plaque volume quantification in the aortic sinus of control and endothelial/macrophage GSK3α/β knockout mice. n = 7–8 (**B**,**C**), * *p* < 0.05, ** *p* < 0.005, *** *p* < 0.0005, ^†^ *p* < 0.05, ^††††^ *p* < 0.00005, ns = not significant.

It was observed that Tie2Cre GSK3α^fl/fl^ LDLR^−/−^ mice have significantly smaller plaque volume, compared to age matched GSK3α^fl/fl^ LDLR^−/−^ mice (Figure 1C). No significant difference was detected between Tie2Cre GSK3β^fl/fl^ LDLR^−/−^ and GSK3β^fl/fl^ LDLR^−/−^ controls. This suggests that endothelial/macrophage GSK3α is an important contributor to plaque accumulation, and supports previous findings that GSK3α and GSK3β display different functional roles in the context of atherosclerosis [19,21,23,24].

### 2.3. Endothelial Activation Is Reduced in Endothelial/Macrophage Knockout of GSK3α

**Figure 2:**
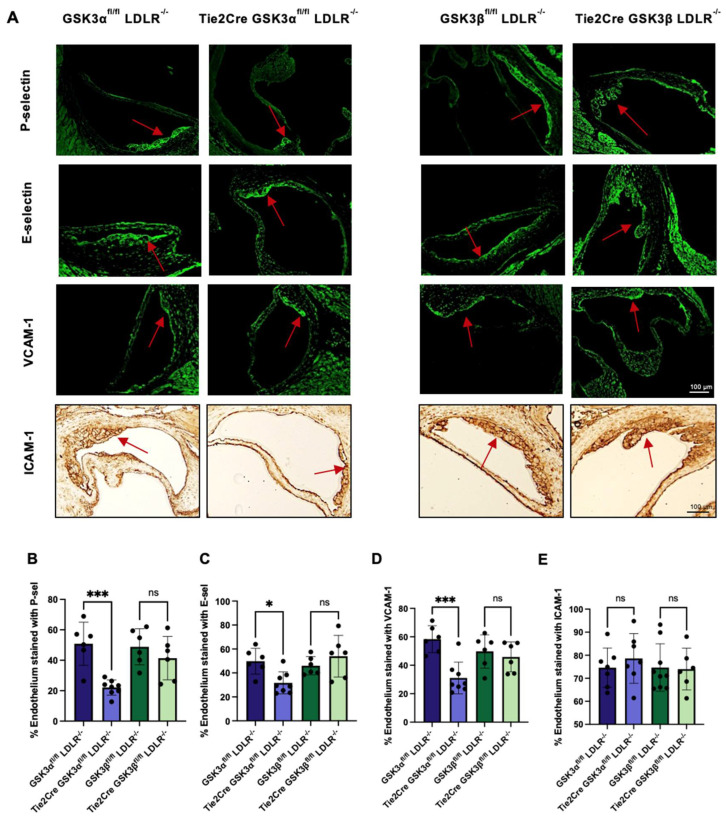
Adhesion protein expression in endothelial/macrophage GSK3α/β knockout mice. (**A**) Representative images of stained aortic sinus cross sections from 15 week old endothelial/macrophage GSK3α/β knockout and control mice. Red arrow indicates atherosclerotic plaque. Scale bar represents 100 μm. Quantification of percentage of endothelium showing immunofluorescent staining of P-selectin (**B**) E-selectin (**C**) and VCAM-1 (**D**). EUR Quantification of immunohistochemistry staining of ICAM-1 expression on the endothelium. *n* = 6–8 (**B**–**E**), * *p* < 0.05, *** *p* < 0.0005. ns = not significant.

EC activation is widely accepted as an important event in monocyte recruitment and the initiation of atherosclerosis [25,26]. To assess endothelial activation, sections of aortic sinus from 15 week old mice were immunostained with antibodies against *p*-selectin, E-selectin, VCAM-1 and ICAM-1. Endothelial staining was verified by co-staining with the endothelial marker, vWF (Supplementary Figure S3). Results indicate that Tie2Cre GSK3α^fl/fl^ LDLR^−/−^ mice have significantly reduced expression of P-selectin, E-selectin and VCAM-1 compared to age matched GSK3α^fl/fl^ LDLR^−/−^ controls (Figure 2B–D). No significant difference was detected in adhesion protein expression between Tie2Cre GSK3β^fl/fl^ LDLR^−/−^ and GSK3β^fl/fl^ LDLR^−/−^ mice. These data are the first indication that GSK3α plays a role in endothelial activation. There was no difference in ICAM-1 expression in any experimental groups (Figure 2E). The specificity of the staining is supported by sections of the aortic sinus stained with appropriate pre-immune IgG in place of the primary antibody (Supplementary Figure S4).

**Figure 3:**
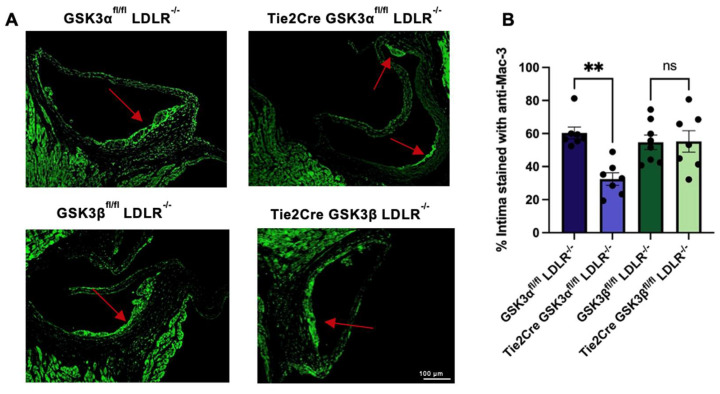
Monocyte/macrophage recruitment in endothelial/macrophage GSK3α/β knockout mice. (**A**) Representative images of cross sections of aortic sinus from 15 week old endothelial/macrophage GSK3α/β knockout and control mice stained with an antibody against Mac-3. Scale bar represents 100 μm. Red arrow indicates atherosclerotic plaque. (**B**) Quantification of percentage of the intima stained with anti-Mac-3 antibody. *n* = 7–8 (**B**), ** *p* < 0.005. ns = not significant.

Sections were stained with an antibody against CD107b^+^/Mac-3^+^ (Mac-3) to assess monocyte/macrophage recruitment. Tie2Cre GSK3α^fl/fl^ LDLR^−/−^ mice had significantly reduced Mac-3 staining, compared to age matched control mice (Figure 3). There was no significant difference in monocyte/macrophage content between Tie2Cre GSK3β^fl/fl^ LDLR^−/−^ and GSK3β^fl/fl^ LDLR^−/−^ control mice, these findings support a role for endothelial and macrophage GSK3α in monocyte/macrophage recruitment.

### 2.4. Bone Marrow Transplant (BMT) Replenished GSK3α and GSK3β Expression in Myeloid Cells

It is possible that the effects on endothelial activation and monocyte/macrophage recruitment, observed above, were an indirect result of macrophage-specific deficiency in GSK3α. A bone marrow transplant was performed to replace myeloid expression of GSK3α and GSK3β, thereby facilitating the assessment of the effects of endothelial GSK3α or GSK3β deficiencies directly.

BMTs were performed on both control and experimental mice. In order to determine if the BMT was successful, DNA was isolated from circulating leukocytes, and the presence of intact genes encoding GSK3α and GSK3β was verified (Supplementary Figure S5A). The expression of GSK3α or GSK3β was confirmed in isolated peritoneal macrophages by RT-PCR analysis (Supplementary Figure S5B). No significant difference in GSK3α or GSK3β expression was detected, suggesting that the BMT was successful. This mouse model will be referred to as the endothelial knockout of GSK3α or GSK3β.

Interestingly, plaques in mice receiving a BMT were significantly smaller than in the mice that did not receive a BMT indicating that the procedure itself did have an effect on atherosclerotic progression (Supplementary Figure S6). Therefore, direct comparisons of atherosclerotic progression in macrophage /endothelial GSK3 knockout mice and BMT endothelial GSK3 knockout mice are not presented.

### 2.5. Endothelial Knockout of GSK3α Attenuates Atherosclerosis

To determine the role of GSK3α or GSK3β in ECs during atherogenesis, bone marrow from 8 week old LDLR^−/−^ mice was transplanted into lethally irradiated 8 week old LDLR^−/−^, Tie2Cre LDLR^−/−^, GSK3α^fl/fl^ LDLR^−/−^, GSK3β^fl/fl^ LDLR^−/−^, Tie2Cre GSK3α^fl/fl^ LDLR^−/−^ and Tie2Cre GSK3β^fl/fl^ LDLR^−/−^ recipient mice. After 4 weeks of recovery, the success of the BMT was verified, and mice were placed on a HFD for 3 weeks. No significant difference was detected in blood glucose, plasma triglycerides, plasma cholesterol, liver weight, adipose weight or pancreas weight between mouse models (Table 2). As previously observed, the body weight of BMT GSK3β^fl/fl^ LDLR^−/−^ mice was significantly higher than controls. Plaque volume of the controls (BMT LDLR^−/−^, BMT Tie2Cre LDLR^−/−^, BMT GSK3α^fl/fl^ LDLR^−/−^ and BMT GSK3β^fl/fl^ LDLR^−/−^) was assessed, and no significant difference was identified between control groups (Supplementary Figure S7), suggesting that Tie2Cre, GSK3α^fl/fl^ or GSK3β^fl/fl^ do not affect plaque development. BMT GSK3α^fl/fl^ LDLR^−/−^ or BMT GSK3β^fl/fl^ LDLR^−/−^ mice were used as controls for the remaining experiments, as they are littermates of the experimental mice.

**Figure 4:**
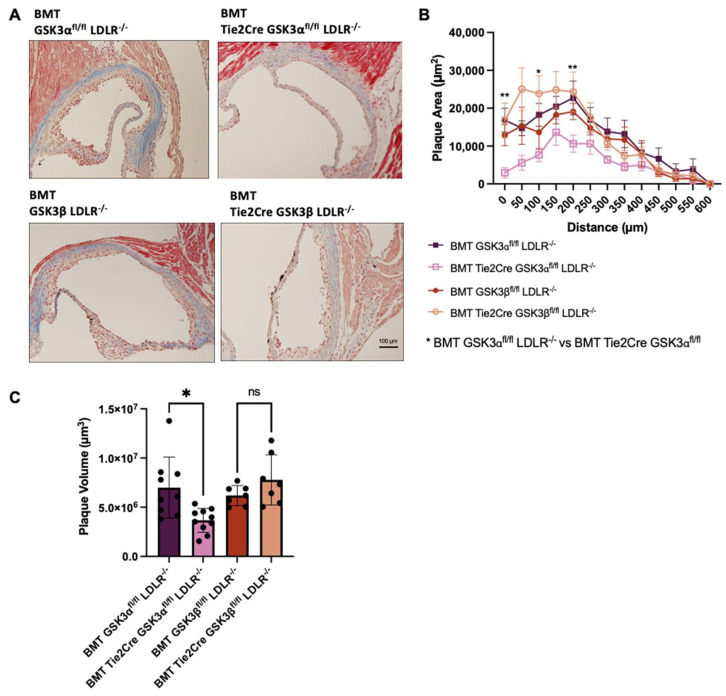
Atherosclerosis in BMT endothelial GSK3α/β knockout mice. (**A**) Representative images of Masson’s trichrome staining of cross sections of aortic sinus from 15 week old BMT control or BMT endothelial GSK3α/β knockout mice. Scale bar represents 100 μm. (**B**) Quantification of plaque area at the aortic sinus of the 15 week old mice. (**C**) Plaque volume quantification in the aortic sinus of BMT control and BMT endothelial GSK3α/β knockout mice. *n* = 7–10 (**B**,**C**), * *p* < 0.05, ** *p* < 0.05. ns = not significant.

To investigate the endothelial specific role of GSK3, plaque volumes of the BMT endothelial GSK3α or GSK3β knockout mice were quantified. Results show that BMT Tie2Cre GSK3α^fl/fl^ LDLR^−/−^ mice have significantly smaller plaques than control mice (Figure 4B,C). No significant difference was seen between BMT Tie2Cre GSK3β^fl/fl^ LDLR^−/−^ and the BMT GSK3β^fl/fl^ LDLR^−/−^ control. This suggests that endothelial GSK3α specifically contributes to atherogenesis and supports previous data that GSK3α and GSK3β play different functions in the context of atherogenesis [19,21,23,24].

### 2.6. Endothelial Activation Is Reduced in Endothelial GSK3α Knockout Mice

**Figure 5:**
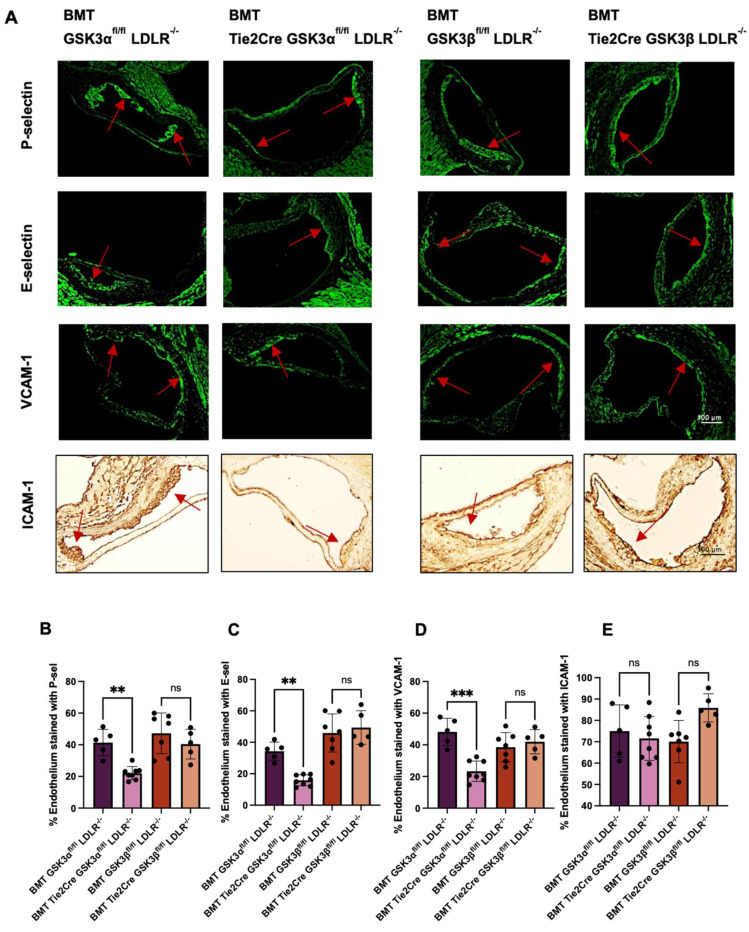
Adhesion protein expression in BMT endothelial GSK3α/β knockout mice. (**A**) Representative images of stained cross sections of aortic sinus from 15 week old BMT endothelial GSK3α/β knockout and BMT control mice. Red arrow indicates atherosclerotic plaque. Scale bar represents 100 μm. Quantification of percentage of endothelium showing immunofluorescent staining of P-selectin (**B**) E-selectin (**C**) and VCAM-1 (**D**). (**E**) Quantification of immunohistochemistry staining of ICAM-1 expression on the endothelium. *n* = 5–8 (**B**–**E**), ** *p* < 0.005, *** *p* < 0.0005. ns = not significant.

To determine the effects that endothelial GSK3α or GSK3β knockout have on endothelial activation, sections of aortic sinus from BMT endothelial knockout mice were immunostained for adhesion markers. BMT Tie2Cre GSK3αfl/fl LDLR^−^/^−^ mice had significantly less staining of P-selectin, E-selectin and VCAM-1 compared to age matched BMT GSK3α^fl/fl^ LDLR^−/−^ controls (Figure 5B–D). No significant difference was detected between BMT Tie2Cre GSK3β^fl/fl^ LDLR^−/−^ and BMT GSK3β^fl/fl^ LDLR^−/−^. There was no difference in ICAM-1 expression in any experimental groups (Figure 5E). These results, combined with the results above, suggest that endothelial GSK3α plays a role in adhesion protein expression thereby affecting/increasing plaque accumulation.

**Figure 6:**
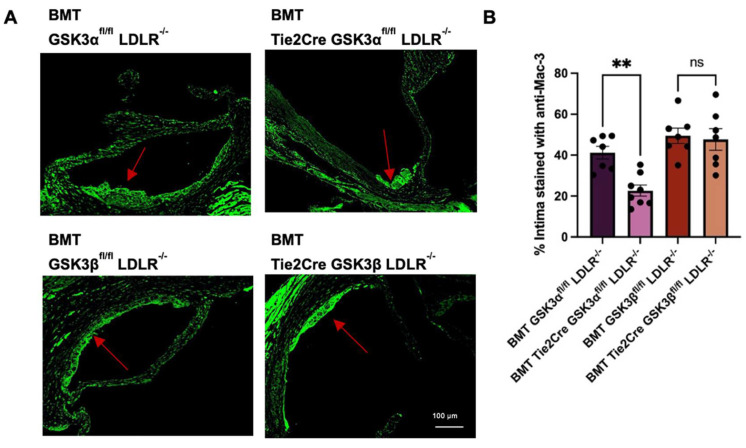
Monocyte/macrophage recruitment in endothelial GSK3α/β knockout mice. (**A**) Representative images of sections of aortic sinus from 15 week old BMT endothelial GSK3α/β knockout and BMT control mice stained with anti-Mac-3. Red arrow indicates atherosclerotic plaque. Scale bar represents 100 μm. (**B**) Quantification of percentage of intima stained with anti-Mac-3 immunofluorescent staining. *n* = 7–8 (**B**). ** *p* < 0.005. ns = not significant.

To assess monocyte/macrophage recruitment, aortic sinus sections were stained with an antibody against Mac-3. BMT Tie2Cre GSK3α^fl/fl^ LDLR^−/−^ had significantly reduced Mac-3 staining, compared to age matched control mice (Figure 6). There was no significant difference in monocyte/macrophage recruitment between BMT Tie2Cre GSK3β^fl/fl^ LDLR^−/−^ and BMT GSK3β^fl/fl^ LDLR^−/−^ control mice. These findings suggest that the effect of endothelial GSK3α knockout on endothelial activation results in a significant reduction in monocyte/macrophage recruitment.

## 3. Discussion

EC activation is believed to be the initiating step in the pathogenesis of atherosclerosis [27]. Activated ECs express adhesion proteins that facilitate the recruitment of circulating monocytes to the artery wall. These monocytes differentiate into macrophages which drive further plaque growth and development. Understanding the mechanism by which cardiovascular risk factors promote atherosclerosis will facilitate the development of therapies to inhibit this process.

In this study we examine the effect of dyslipidemia on the endothelium and specifically focus on the role of GSK3α/β in this process using Tie2Cre recombinase to selectively ablate GSK3 in ECs. As previously noted the Tie2Cre mouse model may not be endothelial specific [22]. Characterization of these novel mouse strains (Tie2Cre GSK3α/β^fl/fl^ LDLR^−/−^) revealed that GSK3α and GSK3β were eliminated in macrophages, as well as ECs. Therefore, this model was used to examine the combined effects of endothelial/macrophage GSK3α or GSK3β knockout. Endothelial/macrophage knockout of GSK3α (Tie2Cre GSK3α^fl/fl^ LDLR^−/−^) significantly reduced the expression of endothelial activation markers (P-selectin, E-selectin, VCAM-1) and monocyte/macrophage recruitment, compared to the age matched control (GSK3α^fl/fl^ LDLR^−/−^). ICAM-1 staining showed no difference which has been reported in another atherosclerotic mouse model [28]. Likely as a result of these effects, atherosclerotic plaque size was also significantly reduced in endothelial/macrophage GSK3α knockout mice. These findings indicate that endothelial and/or macrophage GSK3α plays an important role in atherogenesis. Deletion of endothelial/macrophage GSK3β had no significant effect on adhesion protein expression, monocyte/macrophage recruitment, or plaque size. The limitation of these experiments was the inability to clearly distinguish the effects of endothelial from macrophage GSK3α knockout on endothelium activation and atherogenesis.

Previous work from our lab has shown that macrophage knockout of GSK3α does have the effect of reducing atherosclerotic plaque area and volume [21]. Therefore, knocking out GSK3α in the myeloid lineage is likely having an effect on plaque size in this model. To replenish GSK3α in the myeloid lineage, a BMT was performed in order to create an endothelial selective GSK3α or GSK3β knockout mouse model. Results suggest that an endothelial selective GSK3α knockout significantly reduced adhesion protein expression, monocyte/macrophage recruitment, and atherosclerotic plaque area/volume, compared to age matched BMT GSK3α^fl/fl^ LDLR^−/−^ controls. Deletion of endothelial GSK3β had no effect on adhesion protein expression, monocyte/macrophage recruitment, or plaque size, suggesting that endothelial GSK3β may not be an important contributor to atherogenesis. More research is required to assess the possibility of targeting endothelial and/or macrophage GSK3α as a therapy to slow or attenuate atherogenesis. Future studies using small molecule isoform-specific inhibitors for GSK3α [29] will help to test the potential for specific GSK3α inhibition as a therapy to treat CVD.

The downstream signalling pathways through which GSK3α activates pro-atherogenic processes are not known. One potential mechanism may involve activation of NF-κB, resulting in the upregulation of adhesion protein expression. It has been shown that inhibition of GSK3α/β in pancreatic cancer cell (PCC) lines reduces NF-κB activity [30]. Another study using PCC concluded that GSK3α/β maintains NF-κB activity by regulating inhibitory κB kinase activity [31]. It is well established that endothelial NF-κB plays a central role in the regulation of adhesion protein expression [32,33,34,35] as well as the regulation in inflammatory pathways implicated in atherogenesis [36].

This is the first report to indicate a specific role for endothelial GSK3α and further supports the potential of targeting GSK3α as a therapy in the treatment or prevention of atherosclerotic CVD.

## 4. Materials and Methods

### 4.1. GSK3α/β Knockout Mouse Strains

Cre-Lox technology was used to generate mice in which GSK3α or GSK3β is specifically ablated in ECs. LDLR^−/−^ mice (B6.129S7-Ldlrtm1Her/J, Jackson Laboratory, Bar Harbor, ME) were crossed with mice carrying a loxP-flanked GSK3α (GSK3α^fl/fl^) gene or GSK3β floxed (GSK3β^fl/fl^) gene [37,38] to create GSK3α^fl/fl^ LDLR^−/−^ or GSK3β^fl/fl^ LDLR^−/−^ mice. These mice were then crossed with a mouse that expressed Cre recombinase under the control of the endothelial specific Tie2 promoter (B6.Cg-Tg (Tek-Cre)1Ywa/J, Jackson Laboratories, Bar Harbor, ME) to create Tie2Cre GSK3α^fl/fl^ LDLR^−/−^ or Tie2Cre GSK3β^fl/fl^ LDLR^−/−^ mice. All mouse strains existed in a C57Bl/6 genetic background. Validation and characterization of these mice showed that the ablation of GSK3α and GSK3β occurred in ECs, as well as myeloid lineage cells (Supplementary Figure S1). Therefore, these mice were an endothelial/macrophage GSK3α or GSK3β knockout mouse model. To replenish GSK3α or GSK3β in the myeloid lineage, bone marrow from LDLR^−/−^ mice was transplanted into GSK3α^fl/fl^ LDLR^−/−^, GSK3β^fl/fl^ LDLR^−/−^, Tie2Cre GSK3α^fl/fl^ LDLR^−/−^, Tie2Cre GSK3β^fl/fl^ LDLR^−/−^, or LDLR^−/−^ (control) mice (see below). These mice were used as endothelial-selective GSK3α or GSK3β knockout mouse models.

### 4.2. Bone Marrow Transplant

Eight week old LDLR^−/−^, Tie2Cre LDLR^−/−^, GSK3α^fl/fl^ LDLR^−/−^, GSK3β^fl/fl^ LDLR^−/−^, Tie2Cre GSK3α^fl/fl^ LDLR^−/−^ and Tie2Cre GSK3β^fl/fl^ LDLR^−/−^ mice were irradiated with 667 RAD and then 333 RAD, 3 h later, for a total dose of 1000 RAD. Irradiated recipient mice were injected with bone marrow harvested from the tibias and femurs of 8 week old donor LDLR^−/−^ mice (1 × 10^6^ cells/recipient). The recipient mice were allowed to recover for 4 weeks, during which they received mush food combined with Nutri-cal (Vetoquinol, France) and hydrogel. The bone marrow transplant was validated by isolating DNA from circulating leukocytes using a DNeasy Blood and Tissue Kit (Qiagen, Hilden, Germany) and RT-PCR analysis of peritoneal macrophages (Primers Table 3) (Supplementary Figure S5A,B).

### 4.3. Experimental Design

At 12-weeks of age female mice (LDLR^−/−^, Tie2Cre LDLR^−/−^, GSK3α^fl/fl^ LDLR^−/−^, GSK3β^fl/fl^ LDLR^−/−^, Tie2Cre GSK3α^fl/fl^ LDLR^−/−^ and Tie2Cre GSK3β^fl/fl^ LDLR^−/−^), both endothelial /macrophage GSK3α or GSK3β knockout mice and BMT endothelial GSK3α or GSK3β knockout mice, were placed on a high-fat diet ((HFD) containing 21% fat, 0.2% cholesterol with 42% of the calories from fat, Harland Tekland, TD97363) for 3-weeks to establish atherosclerotic plaques. Mice were maintained on a 12 h light/dark cycle with unlimited access to food and water. All mice were harvested at 15-weeks of age (Supplementary Figure S8). All experiments were performed in female mice because 3 weeks of high fat diet feeding produced only minimal lesions in male mice. Littermates lacking Cre recombinase were used as controls. Additional controls include LDLR^−/−^ mice and Tie2Cre LDLR^−/−^ mice. Experiments were performed according to the guidelines and regulations of the Canadian Council on Animal care and all animal studies were pre-approved by McMaster University Animal Research Ethics Board.

### 4.4. Tissue Harvesting

Mice were anesthetized and a midline laparotomy incision was made. PBS was flushed through the apex of the heart to rinse the vasculature. The whole aorta was carefully removed and cleaned from surrounding muscle and adventitial fat. Other tissues included heart, lung, liver, and skeletal muscle were also removed from some mice for further analysis.

### 4.5. EC Isolation

ECs were isolated using a protocol adapted from Chen S. et al. [39]. Whole aortas were longitudinally dissected, and the tunica intima side of the vessel was washed in 50 μL of 0.25% Trypsin (Gibco, Waltham, MA) for 2 min at 37 °C in 5% CO_2_. ECs were collected and placed in 10mL media (EGM™-2 Endothelial Cell Growth Medium-2 BulletKit (Lonza, Basel, Switzerland) supplemented with 1% penicillin-streptomycin). This process was repeated 4 times. The collected cells were centrifuged at 500 rpm for 10 min, the supernatant was discarded, and cells were resuspended in TRIzol Reagent (Thermo Fisher Scientific, Waltham, MA, USA). The identity of the isolated cells was confirmed by assessing vWF gene expression by RT-PCR (Supplementary Figure S1C).

### 4.6. Macrophage Collection

To collect peritoneal macrophages, 1mL of thioglycolate (10%, Sigma-Aldrich, St. Louis, MO, USA) was injected into the peritoneal cavity. After 5 days, the mouse was euthanized, and 10 mL of 0.05 mM EDTA-PBS was injected into the peritoneal cavity. The peritoneum was massaged to achieve maximum yield, and the macrophages were collected using a 10 mL syringe. Macrophages were centrifuged and resuspended in DMEM media (supplemented with 20% FBS, 1% pen strep and 1% L-glutamine) and plated in a 6 well plate. After 2 h the plates were washed 3 times with PBS, to remove red blood cells and other debris. Macrophage RNA was isolated using the TRIzol Reagent method (Thermo Fisher Scientific, Waltham, MA, USA).

### 4.7. Assessing Tissue Specific GSK3α and GSK3β Gene Expression

Total RNA was isolated from peritoneal macrophages, lung, liver, skeletal muscle and aortic ECs using the TRIzol reagent method (Thermo Fisher Scientific, Waltham, MA, USA) and cDNA was synthesized using the High-Capacity cDNA Reverse Transcription Kit (Thermo Fischer Scientific, Waltham, MA, USA). The expression of GSK3α and GSK3β was quantified using the SYBR Green (with ROX) method (Thermo Fischer Scientific, Waltham, MA, USA) and the StepOnePlus Real-Time PCR System (Thermo Fischer Scientific, Waltham, MA, USA) (Primers Table 1). Samples were run in technical triplicates. The comparative Ct method was used to determine relative gene expression and expression levels were normalized to a β-actin control.

### 4.8. Analysis of Blood and Plasma

Mice were fasted for 4 h. Fasting blood glucose levels were measured using a OneTouch Verio Flex Meter (Lifescan, Inc., Milpitas, CA, USA), fasting plasma total cholesterol and triglyceride levels were determined using the colormetric Infinity Cholesterol or Infinity Triglyceride Kit (Thermo Fisher Scientific, Waltham, MA, USA). Assays were performed according to the manufacturer’s instructions.

### 4.9. Atherosclerotic Plaque Analysis

Hearts were embedded in paraffin and 5 μm thick serial sections of the aortic sinus were collected on glass slides [40]. Sections were deparaffinised and then stained with Weigert’s Hematoxylin (Sigma-Aldrich, St. Louis, MI, USA). The slides were further stained with Biebrich scarlet-acid fuchsin solution and alanine blue (Sigma-Aldrich, St. Louis, MI, USA). Coverslips were attached using DPX mounting media and images were captured using the Olympus DP71 digital camera (Olympus imaging, Centre Valley, PA, USA) mounted on a Leitz Laborlux S bright-field microscope (Leica Microsystems, Concord, ON, USA). Atherosclerotic plaque areas were determined from 12 serial sections per mouse. The atherosclerotic volumes were determined using ImageJ software (Version1.15j8, https://imagej.nih.gov/ij/index.html (accessed on 8 November 2022)), as previously described [40].

### 4.10. Analysis of EC Activation

Endothelial activation was assessed through immunofluorescent and immunohistochemical analyses. Sections of aortic sinus were immunostained with antibodies against P-selectin (Novus Biologicals, Littleton, CO, NB100-65392 1:50 dilution), E-selectin (Novus Biologicals, Littleton, CO, USA, NBP1-45545 1:100 dilution), VCAM-1 (Abcam, Cambridge, UK, ab134047 1:50 dilution), vWF (Agilent Dako, Santa Clara, CA, USA, A008202 1:50 dilution or Proteintech, Rosemount, IL, USA, 66682-1-Ig, 1:100) and CD107b+/Mac3 (BD Biosciences, Franklin Lakes, NJ, USA, 550292 1:50 dilution). Primary antibodies were detected with appropriate conjugated secondary antibodies (Alexa Fluor 488 anti-mouse, Alexa Fluor 488 anti-rabbit or Alexa Fluor 488 anti-rat–all from Thermo Fisher Scientific, Waltham, MA, USA) and the percentage of the endothelium stained in each case was determined by immunofluorescence. Immunohistochemistry was used to assess ICAM-1 expression, using a primary antibody (R&D Systems, Minneapolis, MN, USA, BAF796 1:50 dilution) and the appropriate biotinylated anti-goat secondary antibody (Thermo Fisher Scientific, Waltham, MA, USA). Horseradish peroxidase and 3,3′ diaminobenzidine (DAB) (Agilent, Santa Clara, CA, USA) was used to detect immunohistochemical staining. Negative controls for staining were performed using pre-immune IgG in place of the primary antibody (Supplementary Figure S4).

### 4.11. Statistical Analysis

Analysis of data was performed on Graph Pad Prism 9 and analyzed using a *t*-test, one or two-way ANOVA test followed by a multiple comparison test. All error bars on graphs represent the standard error of the mean (+/− SEM). For all experiments, a *p* value of < 0.05 was considered to be statically significant. * *p* < 0.05, ** *p* < 0.005, *** *p* < 0.0005, **** *p* < 0.00005.

## Figures and Tables

**Table 1:** Metabolic Parameters for endothelial/macrophage knockout mice.

Genotype	Blood Glucose (mM)	Plasma Triglycerides (mM)	Plasma Cholesterol (mM)	Body Weight (g)	Liver Weight (g)	Adipose Weight (g)	Pancreas Weight (g)
LDLR^−/−^	10.85 ± 0.81	2.30 ± 0.22	11.23 ± 0.42	22.71 ± 0.97	1.30 ± 0.16	0.31 ± 0.06	0.13 ± 0.01
Tie2Cre LDLR^−/−^	9.39 ± 0.37	2.12 ± 0.18	11.11 ± 0.16	20.40 ± 0.78	1.00 ± 0.05	0.18 ± 0.03	0.11 ± 0.00
GSK3α^fl/fl^ LDLR^−/−^	9.56 ± 0.61	2.06 ± 0.12	10.18 ± 0.25	22.61 ± 0.77	1.11 ± 0.07	0.22 ± 0.03	0.12 ± 0.00
Tie2Cre GSK3α^fl/fl^ LDLR^−/−^	9.86 ± 0.85	2.43 ± 0.19	10.38 ± 0.41	21.56 ± 0.55	1.04 ± 0.08	0.22 ± 0.02	0.13 ± 0.01
GSK3β^fl/fl^ LDLR^−/−^	10.92 ± 1.35	2.71 ± 0.52	11.39 ± 0.29	27.35 ± 0.58 **^/††††^	1.27 ± 0.05	0.48 ± 0.15 ^††^	0.14 ± 0.01
Tie2Cre GSK3β^fl/fl^ LDLR^−/−^	10.75 ± 1.26	4.33 ± 0.56 *^/†^	11.44 ± 0.43	25.55 ± 1.47 **^/††††^	1.16 ± 0.17	0.24 ± 0.06 ^†^	0.16 ± 0.00

* *p* < 0.05 and ** *p* < 0.005 compared to LDLR^−/−^; ^†^ *p* < 0.05, ^††^ *p* < 0.005, ^††††^ *p* < 0.00005 compared to Tie2Cre LDLR^−/−^; *n* = 7–9.

**Table 2:** Metabolic Parameters for BMT endothelial knockout mice.

Genotype	Blood Glucose (mM)	Plasma Triglycerides (mM)	Plasma Cholesterol (mM)	Body Weight (g)	Liver Weight (g)	Adipose Weight (g)	Pancreas Weight (g)
BMT LDLR^−/−^	9.6 ± 0.16	1.99 ± 0.22	9.55 ± 0.21	19.54 ± 0.72	0.89 ± 0.04	0.16 ± 0.06	0.12 ± 0.05
BMT Tie2Cre LDLR^−/−^	10.15 ± 1.14	2.67 ± 0.27	8.89 ± 0.51	21.93 ± 0.34	1.33 ± 0.20	0.23 ± 0.01	0.31 ± 0.23
BMT GSK3α^fl/fl^ LDLR^−/−^	11.54 ± 0.88	1.97 ± 0.15	8.25 ± 0.28	25.36 ± 1.43	1.29 ± 0.09	0.28 ± 0.06	0.10 ± 0.01
BMT Tie2Cre GSK3α^fl/fl^ LDLR^−/−^	12.08 ± 0.60	1.63 ± 0.12	7.71 ± 0.26	21.56 ± 0.82	1.13 ± 0.10	0.18 ± 0.03	0.09 ± 0.01
BMT GSK3β^fl/fl^ LDLR^−/−^	11.60 ± 0.56	1.58 ± 0.22	7.01 ± 0.34	28.12 ± 1.51 **^/†/‡‡^	1.41 ± 0.15	0.42 ± 0.19	0.12 ± 0.01
BMT Tie2Cre GSK3β^fl/fl^ LDLR^−/−^	10.20 ± 0.49	2.02 ± 0.24	7.31 ± 0.33	22.40 ± 0.70	1.00 ± 0.07	0.18 ± 0.02	0.09 ± 0.00

** *p* < 0.005 compared to BMT LDLR^−/−^; ^†^ *p* < 0.05 compared to BMT Tie2Cre LDLR^−/−^; ^‡‡^ *p* < 0.005 compared to BMT Tie2Cre GSK3β^fl/fl^ LDLR^−/−^; *n* = 7–10.

**Table 3:** Primer sequences.

Transcript	Primer Sequence
GSK3α	F: 5′- GAG CGT TCC CAA GAA GTG G -3′ R: 5′- GTG CCT GGT ATA CTA CTC CGA -3′
GSK3β	F: 5′- ATA AAG ATG GCA GCA AGG TAA CCA -3′ R: 5′- CTG ACT TCC TGT GGC CTG TCA -3′
vWF	F: 5′-GCA GTG GAG AAC AGT GGT G -3′ R: 5′- GTG GCA GCG GGC AAA C -3′
β-actin	F: 5′- GGG GTG TTG AAG GTC TCA AAC -3′ R: 5′-GGC ACC ACA CCT TCT ACA ATG -3′

## Data Availability

Not applicable.

## Supplementary Materials

The following supporting information can be downloaded at: https://www.mdpi.com/article/10.3390/ijms232314780/s1.
