# Supplementary material for: Investigating the Role of Endothelial Glycogen Synthase Kinase3α/β in Atherogenesis in Low Density Lipoprotein Receptor Knockout Mice
Source: Int J Mol Sci. 2022 Nov 26;23(23):14780. doi: 10.3390/ijms232314780 (PMC9740237; doi:10.3390/ijms232314780)
Supplement: Supplementary file 1 [file ijms-23-14780-s001.zip › ijms-2055819-supplementary.pdf]

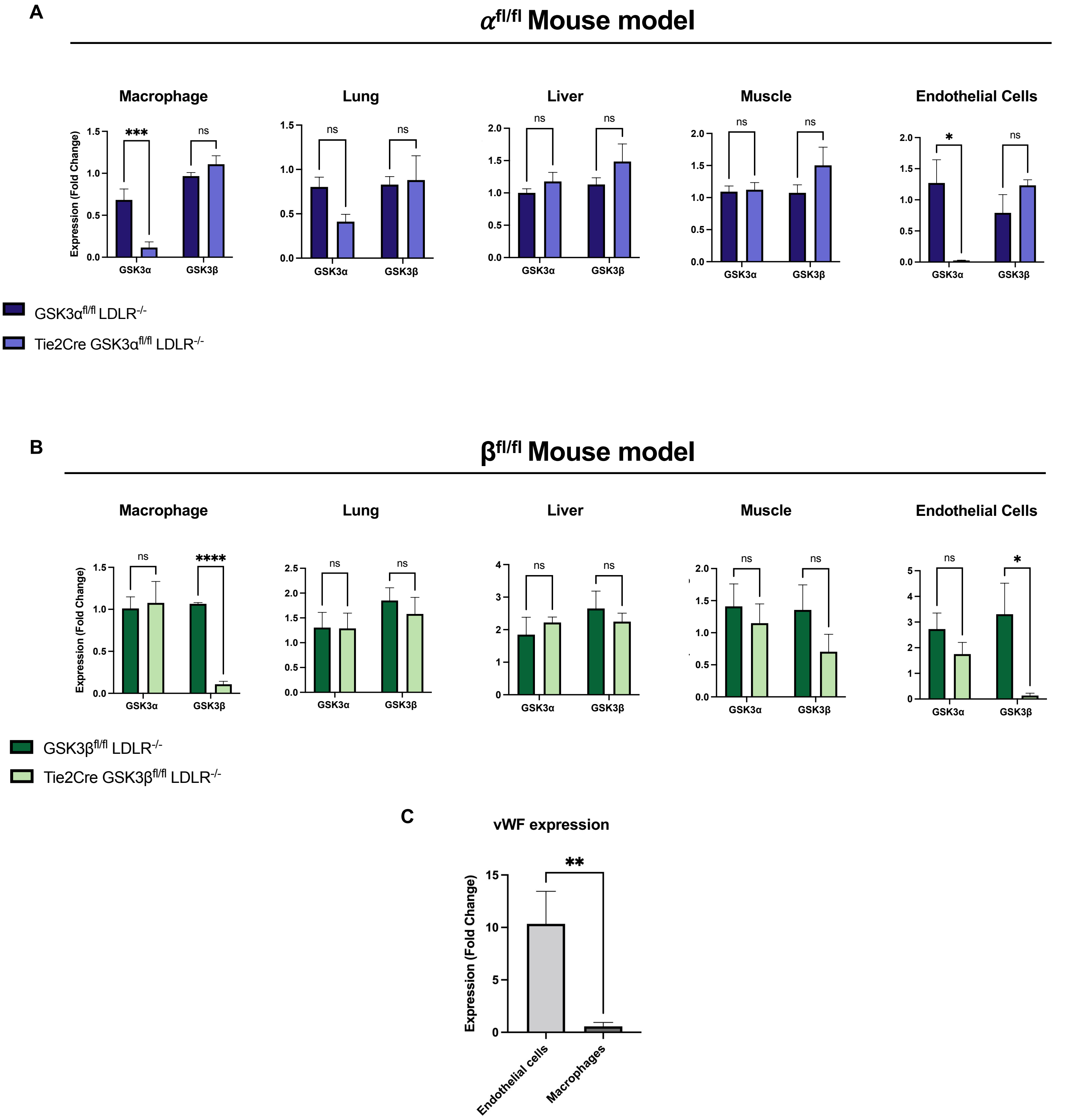

Supplementary Figure S1: ***Characterizing the endothelial/macrophage GSK3α/β knockout mouse model.*** **A** Gene expression of GSK3α/β was assessed in macrophages, lung, liver, skeletal muscle and endothelial cells from GSK3$\alpha^{fl/fl}$ $LDLR^{-/-}$ and Tie2Cre GSK3$\alpha^{fl/fl}$ $LDLR^{-/-}$ mice. **B** Gene expression of GSK3α/β was assessed in macrophages, lung, liver, skeletal muscle and endothelial cells from GSK3β $^{fl/fl}$ $LDLR^{-/-}$ and Tie2Cre GSK3$\alpha^{fl/fl}$ $LDLR^{-/-}$ mice. Gene expression was normalised to a βactin control. **C** To verify that the cells isolated from murine aortas were, in fact, endothelial cells, the expression of endothelial marker, vWF was assessed. n=3-7, * $P<0.05$, **$P<0.005$, ***$P<0.0005$, ****$P<0.00005$.

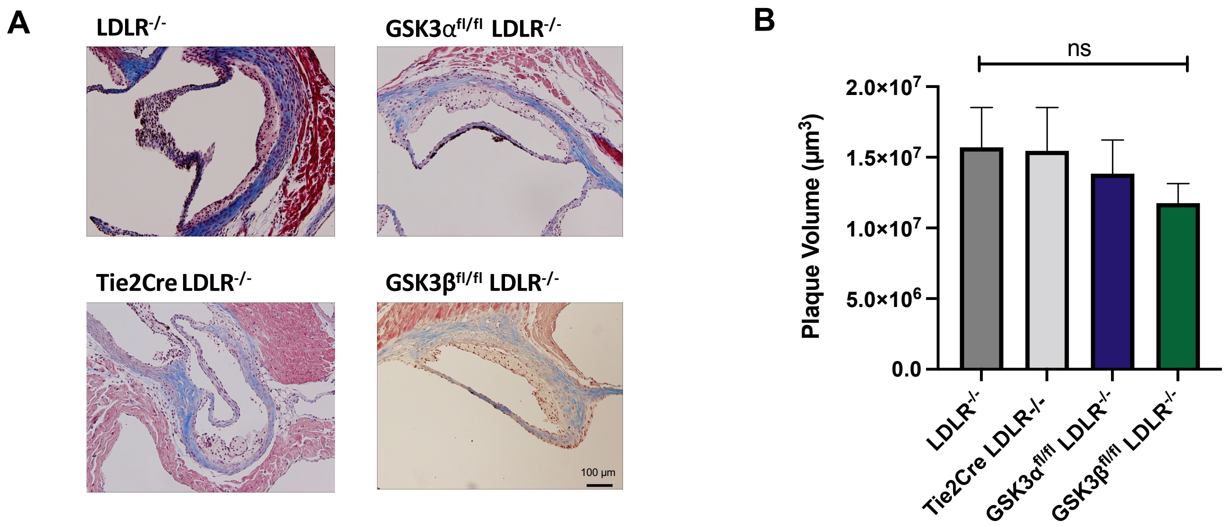

Supplementary Figure S2: ***Atherosclerosis in control mice from the endothelial/macrophage GSK3α/β knockout mouse model.*** **A** Representative images of aortic sinus cross sections from 15 week old control LDLR$^{-/-}$, Tie2Cre LDLR$^{-/-}$, GSK3α$^{fl/fl}$ LDLR$^{-/-}$ and GSK3β$^{fl/fl}$ LDLR$^{-/-}$ mice stained with Masson's Tricrhome. Scale bar represents 100μm. **B** Plaque volume quantification in the aortic sinus of control mice, n=4-8.

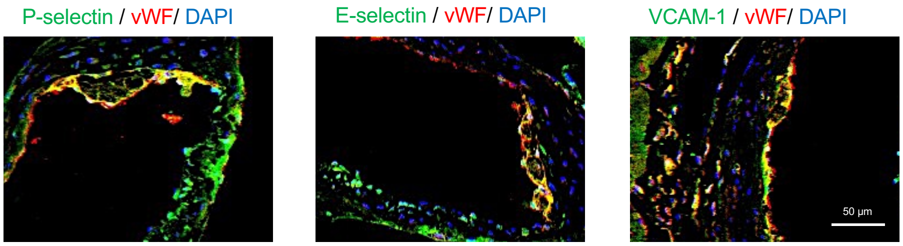

Supplementary Figure S3: ***Co-staining of adhesion protein and vWF.*** Representative images of aortic cross sections stained with antibodies against vWF and P-selectin, E-selectin or VCAM-1. Green represents the adhesion proteins (P-selectin, E-selectin and VCAM-1), red represents vWF staining and blue represents DAPI. Yellow represents where both proteins are present. Scale bar represents 50µm.

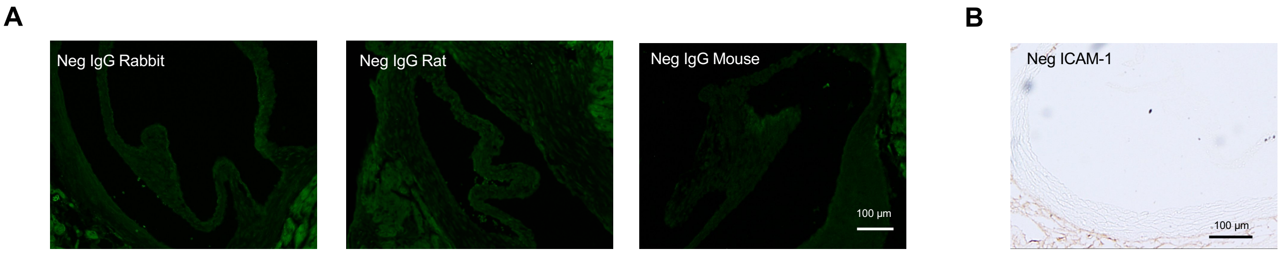

Supplementary Figure S4: ***Negative controls for immunofluorscence and immunohistochemistry staining.*** **A** Pre-immune IgG rabbit was used in place of primary antibody as a negative control for both E-selectin and VCAM-1 staining. Pre-immune IgG rat was used in place of primary antibody as a negative control for Mac-3 staining. Pre-immune IgG mouse was used in place of primary antibody as a negative control for P-selectin staining. **B** Pre-immune goat IgG was used in place of the primary antibody for a negative control for ICAM-1 staining. Scale bar represents 100µm.

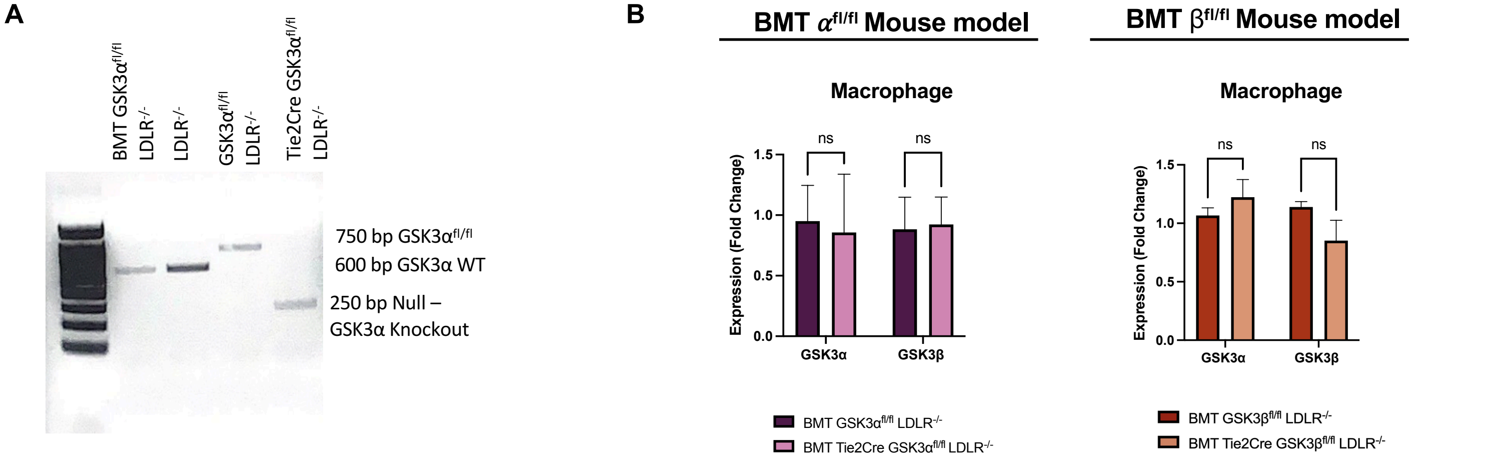

Supplementary Figure S5: ***Confirmation that BMT restored GSK3α and GSK3*****β in macrophages. A** PCR analysis of genomic DNA from blood cells from BMT GSK3α$^{fl/fl}$ LDLR$^{-/-}$, LDLR$^{-/-}$, GSK3α$^{fl/fl}$ LDLR$^{-/-}$, and Tie2Cre GSK3α$^{fl/fl}$ LDLR$^{-/-}$ mice. Results confirm that BMT mice have the GSK3α wildtype (WT) gene restored. **B** RT-PCR analysis of GSK3α/β expression in macrophages isolated from BMT GSK3α$^{fl/fl}$ LDLR$^{-/-}$, BMT Tie2Cre GSK3α$^{fl/fl}$ LDLR$^{-/-}$, BMT GSK3β$^{fl/fl}$ LDLR$^{-/-}$, and BMT Tie2Cre GSK3β$^{fl/fl}$ LDLR$^{-/-}$ mice after a BMT, n=3.

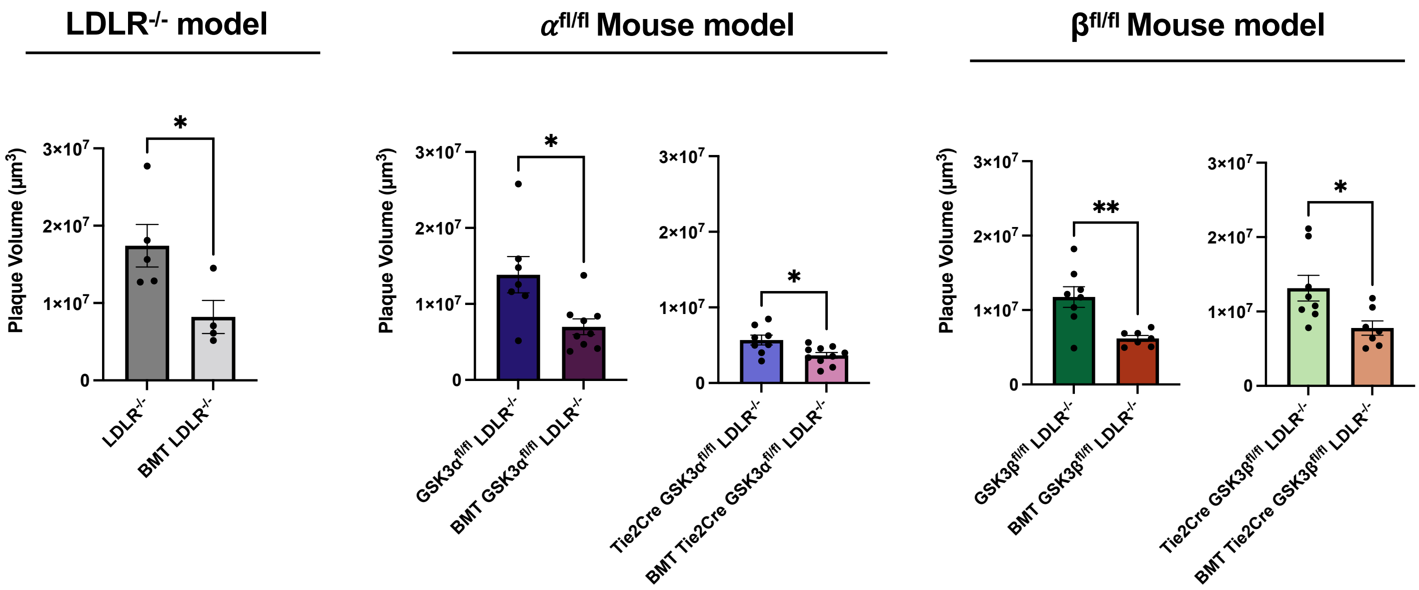

Supplementary Figure S6: ***Atherosclerosis assessment comparison between non-BMT mice and BMT mice.*** Plaque volume quantification in the aortic sinus of non-BMT mice and BMT mice in LDLR$^{-/-}$ mice, $\alpha^{fl/fl}$ mouse model and the $\beta^{fl/fl}$ mouse model. n=4-10, *<P 0.05, **<P0.05.

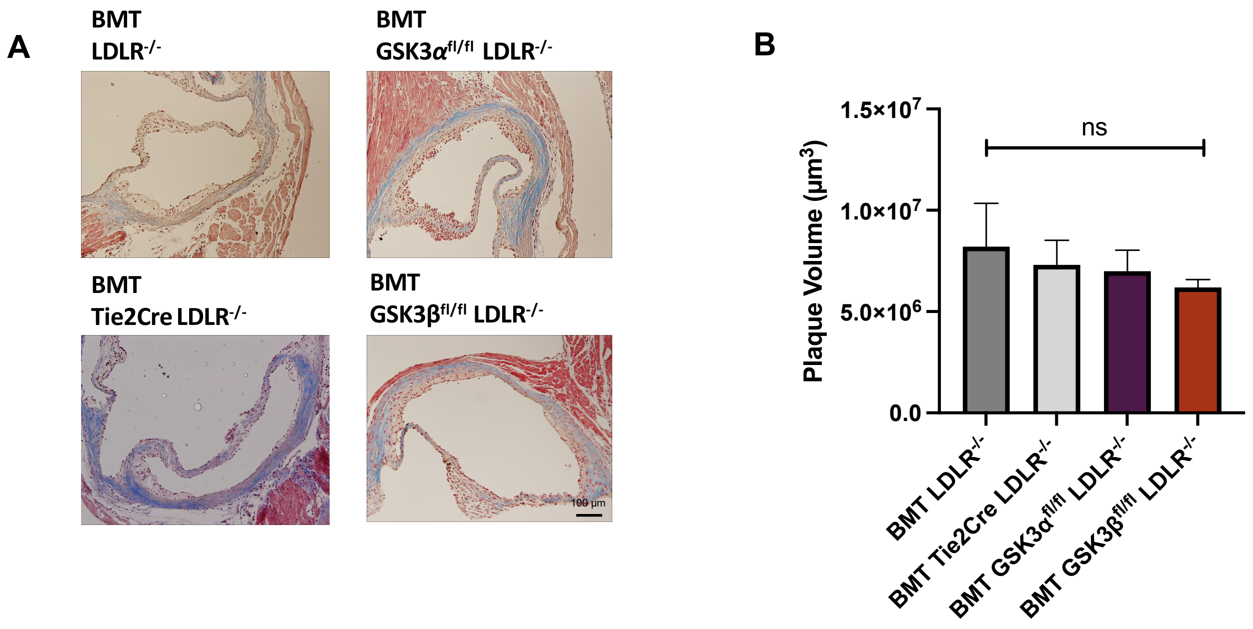

Supplementary Figure S7: ***Atherosclerosis assessment in control mice from the BMT endothelial GSK3α/β knockout mouse model.*** **A** Representative images of Masson's Tricrhome staining of aortic sinus cross sections from 15 week old control BMT LDLR$^{-/-}$, BMT Tie2Cre LDLR$^{-/-}$, BMT GSK3α$^{fl/fl}$ LDLR$^{-/-}$ and BMT GSK3β$^{fl/fl}$ LDLR$^{-/-}$ mice. Scale bar represents 100µm. **B** Plaque volume quantification in the aortic sinus of control mice, n=4-9.

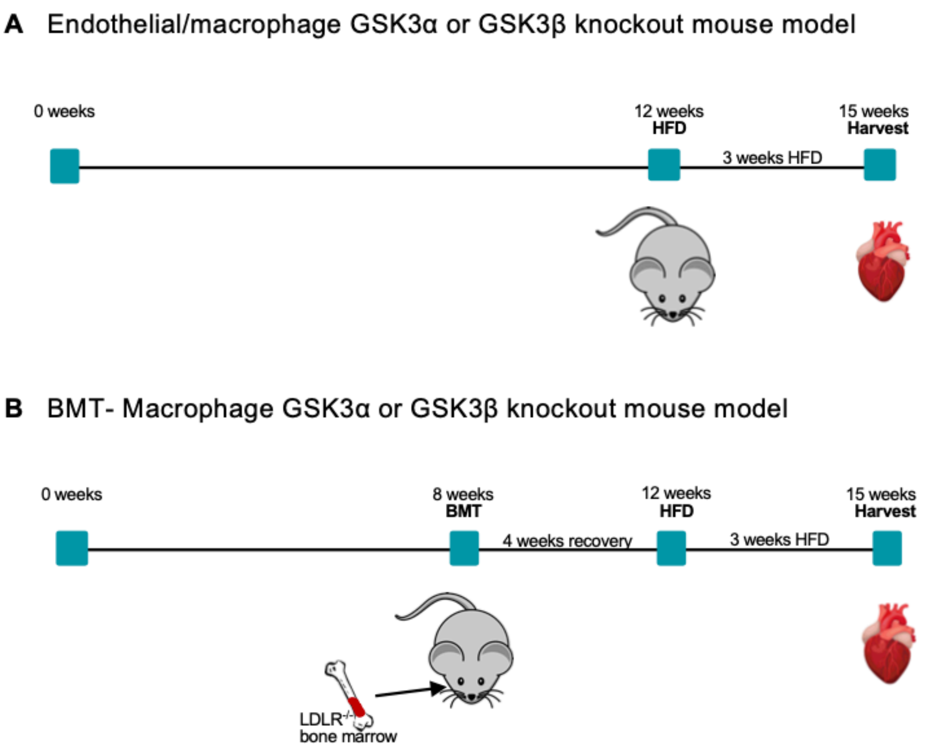

Supplementary Figure S8: ***Experimental design.*** **A** Experimental plan for analysis of endothelial/macrophage GSK3 knockout mice. Genotyped mice were placed on a HFD for 3 weeks and harvested at 15 weeks of age. **B** Experimental plan for BMT endothelial GSK3α/β knockout mice. Genotyped mice received a BMT at 8 weeks of age. After 4 weeks of recovery the mice were placed on a HFD for 3 weeks and harvested at 15 weeks of age. *Figure created using Biorender.com 2022.*
